# Memoir on the Field Carriage of Sick and Wounded Soldiers in the Bengal Army

**Published:** 1846-07

**Authors:** 


ART. I.—*Memoir on the Field Carriage of Sick and Wounded Soldiers in the Bengal Army.* By J. S. LOGIN, M.D., Surgeon to the British Residency at Lucknow, Superintendent of H. M. the King of Oude's Hospital, late Surgeon to the Commander-in-Chief in India, &c. &c. Printed at H. M. the King of Oude's Lithographic Press. With coloured drawings.—*Lucknow,* 1844. pp. 36.

THE object of this Memoir is to bring under the notice of the authorities in India a new litter for the transport of sick and wounded soldiers, invented by the author. The objections to the *doolie* at present in use in the Bengal army are pointed out by Dr. Login to be;—the large number of bearers required—the establishment for an European corps of 1000 men being 630—the consequent expense, and the inconvenience of encumbering the army with followers; the inapplicability of the doolie to mountain passes, or to uneven ground; its great bulk; the delay to which this necessarily gives rise in defiling through a narrow pass, or crossing a river in boats; the necessity for having bearers accustomed to the work: these, by existing arrangements, are not under strict discipline as soldiers, and frequently desert at times when their services are most required.

Dr. Login proposes to substitute for the doolie a litter constructed of bamboo, of which he has given several drawings in the Memoir. The advantages it possesses over the common doolie are, that it is little more than half the weight; that it forms an hospital cot, if required; that it can be carried by two men instead of four, or by camels, mules, elephants, ponies, &c., or on carts; when not in use it can be packed up in a small space and carried till required, and in this form could be transported much more rapidly through passes or across rivers.

Dr. Login also proposes that corps of hospital Lascars should be raised to carry these litters, instead of hired doolie-bearers, and who might be employed, when in cantonments, as hospital orderlies and servants, and be required to furnish sentries for the hospital. This arrangement would be productive of saving, compared with the present system, when a regiment took the field; but as this is the exception, and as regiments in India change their quarters, under ordinary circumstances, only once in three years, the expense of keeping up these corps when in cantonments will prove, we fear, a fatal objection. There can be no doubt, however, that such an establishment would be attended with many advantages.

The subject of the transport of sick and wounded soldiers is one of very great importance in India; and at the present time, when it is requisite to assemble such armies as that of the Sutlej, any arrangement for reducing the number of camp followers is deserving of consideration, especially when it promises at the same time to prove beneficial to the soldier. Dr. Login is entitled to great praise for having, in the midst of his other duties, devoted to it so much time and attention. The arguments he has advanced in favour of the proposed system are such as to establish a clear case for affording it a fair trial; and we trust the military authorities in India will give it one on an extensive scale, and under his superintendence.

